# Co-producing mindful tactile technologies for healthy ageing: Cross-cultural insights from India and the UK

**DOI:** 10.1177/20552076251349909

**Published:** 2025-06-17

**Authors:** Madhan Kumar Vasudevan, Ceylan Besevli, Preetham Madapura Nagaraj, Shu Zhong, Manivannan Muniyandi, Marianna Obrist

**Affiliations:** 1 Department of Computer Science, 4919 University College London, London, UK; 2 Department of Applied Mechanics and Biomedical Engineering, 37268 Indian Institute of Technology Madras, India

**Keywords:** Sensory experiences, healthy ageing, cross-cultural, sensory care, touch, mindfulness, well-being

## Abstract

**Objective:**

Healthy ageing and well-being are deeply connected to our sensory experiences, which shape how we interact with the world. As sensory decline is a natural aspect of ageing, it opens opportunities for designing digital technologies that enhance the quality of life. Our objective is to explore how mindful tactile technologies can support healthy ageing by addressing sensory decline in older adults, with a particular focus on cross-cultural insights and user-led design approaches.

**Methods:**

We conducted four co-production workshops: two in India and two in the UK, bringing together older adult attendees from diverse socio-economic backgrounds. These workshops used participatory design methods to gather perspectives on sensory challenges, and emotional well-being and envisioned future technologies related to touch and mindfulness.

**Results:**

Our findings revealed strong connections between sensory experiences, mindfulness, and well-being, as well as distinct cultural differences in how these are experienced and valued. Indian attendees emphasised collective well-being and spiritual practices, while UK attendees focused more on personal autonomy and self-care. These insights informed four key design implications for developing tactile technologies that are culturally adaptive and age-inclusive.

**Conclusion:**

Our work highlights the potential of co-produced, sensory-based digital technologies to enhance the quality of life in older adults. By grounding design in cultural values and lived sensory experiences, our work lays the foundation for future digital health interventions that are both meaningful and inclusive across global ageing populations.

## Introduction

How can we support healthy ageing? This question has long been at the forefront of various research fields focusing on health and well-being. But what if we look at *healthy ageing* through the lens of *sensory experiences*, which form a crucial part of how we interact with and perceive the world? This perspective opens up new avenues for understanding and enhancing the quality of life as we age.

As we age, a decline in sensory capabilities is inevitable.^1–3^ This decline affects our ability to engage with the environment, significantly impacting emotional and overall living experiences. Sensory processing in the human brain underpins every functional task that we perform. Each sensation – whether it be sight, touch, taste, smell, or hearing – has the potential to influence our emotions and overall well-being. The intricate link between sensory and emotional processing in the brain is well-documented in neuroscience literature.^4^

Among the senses, touch stands out as a critical contributor to shaping emotional experiences^5–8^ and fostering higher-order functions such as bonding and pro-social behaviour.^9^ Research shows that tactile acuity, or the ability to perceive distinct tactile stimuli, declines linearly with age.^10^ While assistive devices, such as lenses and hearing amplifiers, can partially address sensory declines in vision and hearing, there are currently no specific enhancers or assistive devices for the senses of touch, smell, or taste.

Cultural factors also play a significant role in how we utilise our sensory experiences. For example, in the Indian subcontinent, eating with fingers is common, allowing people to experience the texture and temperature of food directly.^11^ In contrast, cutlery is predominantly used in Western cultures. Similarly, touch-based social interactions, such as hugging and handholding, are more common in Western cultures than in the Indian subcontinent. Although the biological decline in sensory capabilities due to ageing may be similar across countries, these cultural differences can significantly influence overall sensory experiences and well-being in ageing populations. Therefore, cross-cultural influences must be considered when designing human-centred technologies for healthy ageing.

We conducted four co-production workshops, two in India and two in the UK, to collaboratively understand attendees’ views on healthy ageing and their perspectives on how emerging technology could support mindful touch experiences. Specifically, our focus was on the following questions: (1) What challenges do older adults face in relation to sensory experiences and healthy ageing? (2) How are sensory experiences valued among older adults across different cultures (in India and the UK)? (3) How do older adults envision technologies that support mindful living and tactile sensory engagement? To facilitate these discussions, we introduced workshop attendees to emerging haptic technologies from the field of human–computer interaction (HCI) and mindfulness meditation.

While various technologies are available to support older adults, few are specifically designed to address the sensory experiences critical to healthy ageing. During the workshops, to answer our third question, we introduced attendees to mid-air haptics,^12^ a touchless ultrasound-based technology that has the potential to enhance sensory engagement in everyday life. This technology served as a starting point for our discussions with older adults in both geographic locations. By focusing on being mindful of touch and the sensory world around us, we were able to engage attendees in meaningful conversations about sensory decline and future technological interventions.

This paper presents and reflects upon the outcomes of our co-production workshops in India and the UK, addressing the overarching theme of *global healthy ageing challenges through a sensory experience lens*. Building on key research that advocates supporting HCI research agendas that are shaped by older people,^13^ we pave the way for meaningful and inclusive innovations in this area. Through our workshops, we observed intriguing commonalities and disparities between the ageing communities in the UK and India regarding their perspectives on mindful sensory experiences and well-being. For example, sensory experiences related to spiritual and family bonding were more emphasised by attendees in India, while those in the UK placed greater importance on sensory experiences related to self-care and social support. These findings highlight the dynamic interplay between well-being, mindfulness, and touch experiences. Moreover, they underscore the importance of customisation in technology design, as opposed to a one-size-fits-all approach, to better cater to the diverse needs of older adults across different cultural contexts. We have synthesised our findings into four key design implications^14^ for the HCI community to build upon, extending prior work on mindful technologies and multisensory experiences in ageing, contributing to the design of future technologies that can foster inclusion and support well-being.

### Motivation and cross-cultural focus

The primary motivation for our research was to explore the relationship between sensory experiences, particularly touch, emotional well-being, and ageing through the lens of mindfulness. Since ageing and sensory experiences are perceived differently across cultures, we conducted workshops in two geographically and culturally distinct locations, India and the UK.

Our work represents the first step in a broader cross-cultural dialogue, initiated by the authors’ diverse multicultural backgrounds, which include expertise from India, the UK, China, Turkey, and Italy. Given this diversity, we pragmatically began our exploration with India and the UK, recognising the practical accessibility and cultural relevance these countries provide. The two countries, both part of the Commonwealth, represent Eastern and Western perspectives on ageing, offering distinct sensory engagement profiles. For example, in India, the tactile experience of eating with one’s hands is deeply embedded in cultural practices, while in the UK, touch-based interactions such as handholding are more prominent in social and emotional settings. By selecting these two culturally diverse nations, we aimed to explore how sensory experiences, particularly touch, contribute to emotional well-being and healthy ageing across different cultural contexts. Our long-term goal is to develop a comprehensive understanding of how different societies approach ageing and sensory experiences, ultimately guiding the design of inclusive, culturally sensitive technologies for healthy ageing.

The purpose of our workshops was not to develop a new prototype or conduct a formal study. Instead, we sought to collaboratively explore key topics related to sensory decline, cultural differences in sensory and emotional well-being, and the potential role of mindfulness and emerging technologies, particularly haptic interfaces, in supporting healthy ageing. By engaging attendees in meaningful discussions, we gathered diverse perspectives that will inform the design of future technologies aimed at improving the quality of life for older adults.

## Related works

### Healthy ageing and sensory decline

Ageing is a complex process that affects individuals differently, influenced by a lifetime of social, cultural, and biological factors. Glen Elder’s life course perspective outlines four critical dimensions of ageing: historical context, timing of life events, linked social lives, and human agency.^15,16^ While this framework sheds light on the social dynamics of ageing, it does not fully account for the biological changes, particularly sensory decline, that significantly impact how older adults engage with their environment.

Sensory engagement is crucial for maintaining emotional and physical well-being. Declines in sensory functions, such as touch, vision, and hearing, are common as people age.^1,2,10^ Research indicates that tactile acuity decreases due to neurophysiological changes, including a reduction in mechanoreceptors and slower nervous system response times.^17,18^ This decline makes it harder for older adults to perform simple tasks, such as distinguishing textures, detecting light touch, or sensing temperature changes, impacting their independence and quality of life. Orr et al.^19^ found that older adults frequently describe their sensory experiences with the outdoors, such as the feeling of wind or rain, as integral to their well-being. As such, sensory experiences play a foundational role in how older adults perceive and interact with the world, and addressing sensory decline is essential for promoting healthy ageing.^20,21^

### Sensory engagement and mindfulness meditation

Mindfulness meditation, with its roots in Eastern spiritual traditions, has gained recognition in the West for its benefits on mental and emotional well-being. Studies show that mindfulness practices enhance sensory perception and improve emotional regulation, which can be beneficial for older adults experiencing sensory decline.^22,23^ Mindfulness meditation helps individuals become more aware of subtle sensory experiences, such as the texture of an object or the feel of air on the skin, which may otherwise be overlooked as sensory functions diminish.

Mindfulness has also been shown to alleviate stress, anxiety, and depression,^24,25^ which are common in ageing populations. By heightening awareness and promoting emotional balance, mindfulness can help older adults engage more fully with their sensory environments. For instance, body-scan meditation has been found to improve somatosensory decision-making, encouraging older adults to focus on tactile sensations and potentially offsetting the impact of sensory decline.^22^ Incorporating mindfulness into sensory technologies, particularly haptics, can create immersive experiences that help older adults remain mindful of their sensory surroundings. This integration offers a promising avenue for promoting well-being and engagement in ageing populations, where emotional and sensory decline are closely intertwined.^26^

### Haptic technologies and sensory perception for older adults

Touch is an essential sense for social interaction, emotional connection, and environmental engagement, but it tends to decline with age, leading to reduced tactile acuity and sensitivity.^10,27^ Haptic technologies, which replicate touch sensations through vibration, pressure, or other means, offer a potential solution for enhancing sensory engagement among older adults. These technologies have been widely explored in the context of assistive devices aimed at compensating for sensory loss.

In the field of HCI, there is a growing interest in designing haptic devices that cater specifically to older adults. Baldwin’s work on sensory-cognitive interaction^28^ demonstrates the importance of touch for cognitive functioning and emotional well-being, especially in driving and other critical tasks. Similarly, research by Pang et al.^29^ highlights the need for interfaces that address sensory decline through tactile engagement, emphasising the potential for wearable technologies that can adapt to changing sensory needs. Moreover, wearable haptic devices could enhance emotional well-being by facilitating social connections through tactile feedback. For instance, technologies that mimic the sensation of a hug or handshake could provide emotional comfort, helping to combat loneliness and isolation, common challenges among older adults.^5,7^ These technologies not only address sensory decline but also enrich emotional experiences, fostering a sense of connection and empathy.

Mid-air haptics is an emerging area of research that allows users to experience touchless tactile feedback, offering a promising alternative for older adults with mobility or hygiene concerns.^12,23^ This technology could simulate physical sensations, such as the warmth of sunlight or the pressure of a gentle breeze, without requiring direct contact. Studies have shown that such technologies can support emotional and sensory well-being by recreating immersive, multisensory experiences indoors, which is particularly beneficial for older adults with limited access to outdoor environments.^8^

Designing haptic technologies for older adults, however, requires thoughtful consideration of individual and cultural differences. These technologies must be adaptable and customisable to accommodate varying preferences and needs, ensuring that they can cater to diverse cultural contexts and personal values.^30,31^

### Cross-cultural comparison through theoretical lens

Culture is a complex concept that has been explored in various ways within HCI research. One approach to understanding culture in HCI is through cultural dimensions, such as Hofstede’s model, which is based on cross-cultural psychology.^32^ Hofstede’s model identifies several key dimensions where cultures differ, including power distance, individualism, masculinity, and uncertainty avoidance, and is widely used in cross-cultural HCI studies. This framework can help predict how users from different cultures might interact with technology based on their cultural values. On the other hand, the relationship between HCI and culture is complex, which may explain why detailed cross-cultural methods are not more widely developed. A review found a modest increase in HCI studies considering culture, rising from 0.9% between 1990 and 2005 to 1.9% between 2016 and 2020,^33^ suggesting that culture may still be viewed as a challenge in global interface design.^34^ Another study revealed that nearly 78% of attendees in major HCI studies from 2016 to 2020 came from just four Western countries, while more than half of the world’s countries were underrepresented.^35^ This lack of diversity limits the development of digital health technologies that could address public health challenges in remote and low- to middle-income regions.^36^ In addition, to gain a more nuanced understanding, qualitative methods such as participatory design are also valuable, as they go beyond national or ethnic cultural levels and explore users’ values and traditions more deeply.^37^

### Involving older adults in technology ideation and design

Involving older adults in the ideation and design of technologies is essential to developing solutions that truly meet their needs. Efforts such as co-design^38^ and participatory design^39^ practices have been effective in ensuring that technologies for older adults are user-centred and relevant to their everyday experiences. Co-design, in particular, involves collaboration between users and designers during the design phase, ensuring that the final product reflects the real needs and preferences of the end-users.^31,40^ Participatory design extends this engagement, allowing users to influence the design process more actively by contributing their experiences and insights.

Building on these practices, co-production goes a step further by involving users as equal partners throughout the entire development process, from idea generation to testing and refinement.^41^ Unlike co-design, which primarily focuses on collaboration during the design stage, co-production ensures ongoing involvement, allowing older adults to help shape not only the design but also the development and evaluation of technologies.^42^ This approach is particularly valuable when creating sensory technologies, as it ensures that the solutions are tailored to the lived experiences and evolving needs of older adults.^43^

By engaging older adults through co-production, we can create technologies that are adaptive, customisable, and culturally sensitive. This deeper level of engagement fosters a sense of ownership and ensures that the resulting technologies address not only the physical aspects of ageing but also the emotional and social dimensions, thus supporting diverse ageing populations across the globe.

## Method

In this section, we describe the co-production approach adopted in our research, where older adults and researchers worked together to explore and develop ideas for supporting healthy ageing through a sensory experience lens. Through four co-production workshops held in India and the UK, we engaged attendees in discussions around sensory experiences, ageing challenges, and the role of emerging technologies, particularly mindful touch interfaces, in enhancing well-being. The applied co-production approach does not aim to collect data as in formal user studies, but aims to open up a dialogue with potential target users, in our case elderly, and capture insights from this discussion and observations. In this section, we will guide the reader through our approach and motivation for a cross-cultural perspective on healthy ageing.

The workshops were structured into three progressive phases, each addressing critical aspects of healthy ageing, tactile experiences, and the role of future tactile technologies in supporting sensory well-being and mindfulness for older adults. Specifically, we aimed to answer the following research questions:

1. What challenges do older adults face in relation to sensory experiences and healthy ageing?
2. How are sensory experiences valued among older adults across different cultures?
3. How do older adults envision technologies that support mindful living and tactile sensory engagement?

To provide a clearer understanding of the diversity of attendees across the workshops, we have summarised the main characteristics of attendees involved in this work. Workshop 1 included academic professionals, such as professors and researchers, with extensive experience in higher education and technology use. Workshop 2 involved non-academic attendees from local communities, primarily older adults with varying degrees of exposure to technology. Workshops 3 and 4 in the UK included a balanced mix of older adults from different socio-economic backgrounds, including retirees, community volunteers, and individuals with prior exposure to digital health technologies.

**Figure 1.**
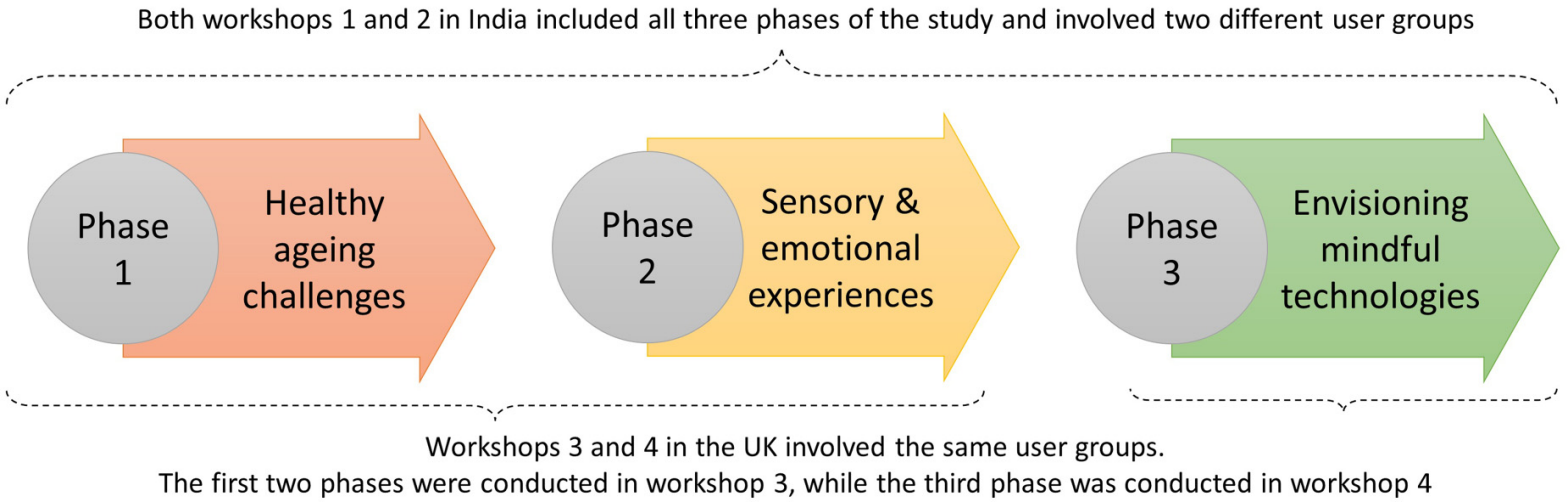
Overview of the three phases followed in the co-production workshops in India and the UK. In Phase 1, attendees explored the challenges of healthy ageing through group discussions, with a focus on sensory decline, cognitive changes, and social isolation. Phase 2 delved deeper into the emotional aspects of sensory experiences, with attendees sharing personal anecdotes about the sensory moments in their daily lives that evoke strong emotional responses. Phase 3 moved towards the future, where attendees were encouraged to brainstorm and conceptualise innovative technologies that could enhance sensory and emotional well-being.

Each workshop was designed to be flexible and responsive to the interests and dynamics of the workshop attendees, ensuring that the process of co-production and ‘making sense together’ took precedence over imposing a fixed structure (see overview in Figure 1). This approach allowed us to explore the topics organically, drawing on the unique insights and lived experiences of the attendees. The following are the three phases (Ph-) followed in the co-production workshops in India and the UK.

**Figure 2.**
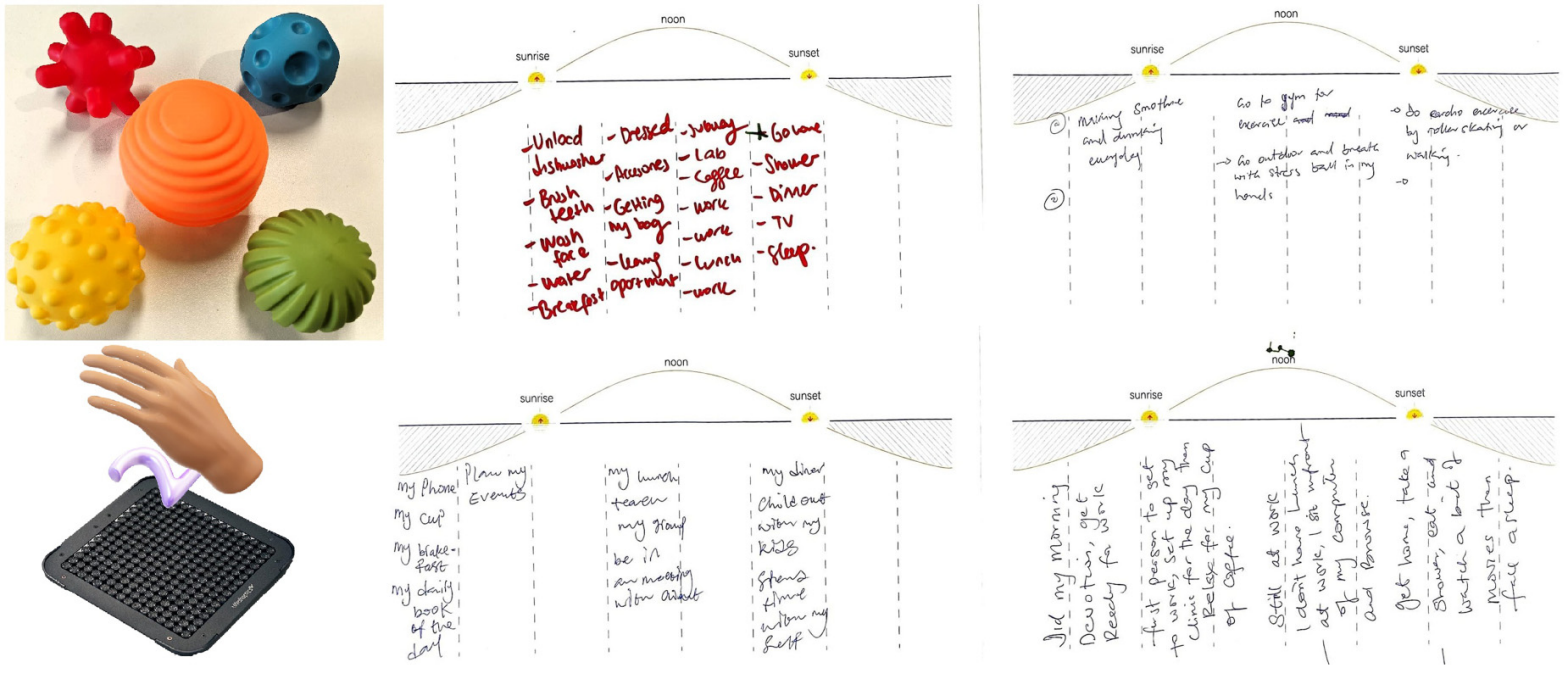
(Top left) During the ice-breaker session, attendees engaged in an activity where they passed balls to one another and described various sensory attributes such as colour, texture, roughness, and softness. Additionally, they were introduced to mid-air haptics technology (bottom left), which demonstrated how future touch technologies might provide contactless tactile sensations. To capture the relationship between daily activities, emotions, well-being, and sensory experiences during phase 2, attendees were asked to document these connections (right: scanned copies) on a piece of paper prepared by the researchers. Pictures are from UK-based workshops.

Ph-1 *Healthy ageing challenges:* The first phase started with an ice-breaker session where attendees introduced themselves while passing balls with unusual spiky surfaces, providing a keyword describing the physical properties (Figure 2, top left). This activity was intended to immerse the attendees in the workshop’s topic of tactile sensations and establish an atmosphere of mutual respect and companionship among the attendees. Afterwards, attendees engaged in facilitated group discussions to unpack the multifaceted challenges linked to healthy ageing. Attendees discussed various aspects such as sensory decline, cognitive changes, physical well-being, and social isolation. Attendees were asked to share their thoughts on these issues, fostering a collective exploration of challenges such as memory loss, emotional well-being, and the importance of social support.
Ph-2 *Sensory and emotional experiences:* In this phase, attendees shared personal experiences that revealed how sensory encounters are deeply intertwined with emotions. To kickstart this process, attendees reflected on their routines and were asked to pinpoint their favourite daily tactile moments, as shown in Figure 2.
Ph-3 *Envisioning mindful technologies:* The final phase focused on brainstorming and conceptualising innovative technologies that could support mindful living and enhance sensory experiences. As a part of this phase, attendees were invited to interact with mid-air haptic technology (Figure 2, bottom left), offering a hands-on introduction to emerging sensory interfaces envisioned for future digital health and well-being tools. Attendees engaged in ideation sessions individually where they imagined future tools to enhance sensory experiences in daily life. Design fiction scenarios were introduced in the workshop held in the UK to stimulate creative thinking. The discussions led to ideas ranging from immersive environments that replicate soothing tactile sensations to technologies that help older adults stay mindful during daily routines.

For our first co-production workshop (W1), held in India, we collaborated with a local university to engage ageing individuals who were retired or still working as academic researchers. Eight attendees, aged between 50 and 95 years (1 female, 7 male), attended this workshop. All attendees were familiar with multisensory interaction technologies and experienced as professors in various research fields. This group was selected to leverage their academic expertise and focus on the theoretical and technical aspects of sensory decline and ageing. The goal of W1 was to explore the intersection of sensory experiences, ageing, and technological innovations from a scholarly perspective, drawing on the attendees’ research backgrounds.

For the second co-production workshop (W2), we shifted our focus to engaging a more diverse group of elderly individuals from an elderly community club. Twenty-four retired professionals from various non-academic backgrounds, aged between 60 and 96 years (6 female, 18 male), attended this workshop. The purpose of W2 was to gather a broader range of perspectives from individuals with practical life experiences in different fields, thereby complementing the insights gathered from the first workshop. Given the larger group size, the structure of W2 was modified to facilitate more focused discussions by dividing the attendees into six smaller groups.

By conducting these two workshops with different attendee profiles, we aimed to capture a more holistic understanding of healthy ageing and sensory experiences. W1 focused on theoretical and research-driven discussions, while W2 allowed us to explore these themes in the context of everyday lived experiences. The diversity of the attendees across both workshops enabled us to derive richer insights and more comprehensive design implications for supporting sensory well-being in older adults.

For the third co-production workshop (W3), held in the UK, we collaborated with a local co-production agency for recruitment. Eight attendees, aged between 45 and 70 years (3 female, 5 male), from diverse professional backgrounds participated in this workshop. Unlike the workshops conducted in India, where all three phases were covered in a single session, the structure for the workshops held in the UK was adapted based on feedback and observations from earlier workshops. In India, attendees progressed through all phases in a single session, which provided a continuous flow but limited the depth of discussion in each phase. Therefore, in the UK, we decided to divide the workshop into two parts: phases 1 and 2 were addressed in one session (W3), while phase 3 was explored in a separate follow-up session (W4). This change was made to allow for a deeper exploration of the themes and to provide attendees with more time to reflect on their experiences before moving on to envisioning future technologies.

The iteration of the workshop structure also took into account the cultural and contextual differences between the Indian and UK attendees. In the UK, we dedicated more time to familiarise attendees with the concept of mindfulness which was provided by the lead researcher in the first workshop as a guided meditation session. As a result, breaking the workshop into two parts facilitated a more gradual and thorough engagement with these concepts. The following subsections elaborate on these structural adjustments and their outcomes.

**Figure 3.**
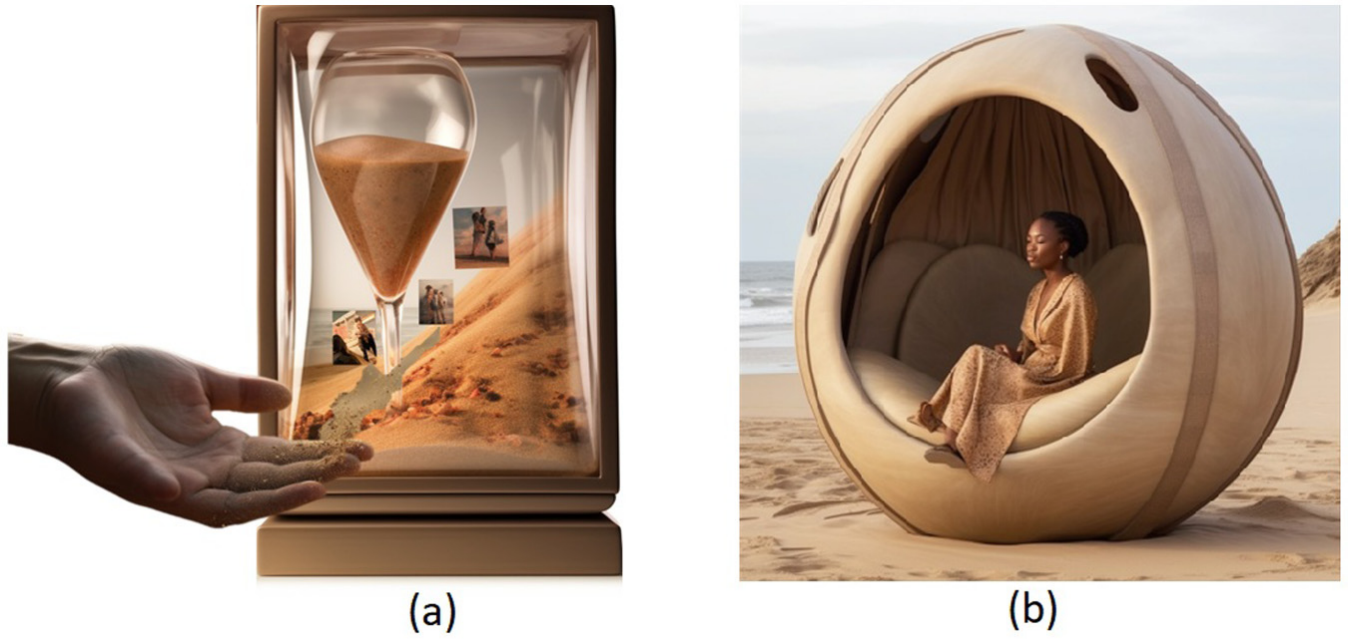
Scenarios developed in Step 1 of W3 by HCI researchers. (a) Memory Hourglass: a tabletop box that offers tactile and immersive experiences through cherished memories and (b) Immersive Oasis: a special pod that takes users to different places through touch and other sensations. Images generated by authors using the artificial intelligence tool Midjourney. W3: third co-production workshop; HCI: human–computer interaction.

W4 focused on phase 3, where the aim was to envision future technologies that could support sensory experiences and emotional well-being. To start off the ideation process, we introduced two design fiction^44^ scenarios, which explore how potential users perceive and interpret future technologies.^45^ The decision to use design fiction scenarios in this workshop was informed by observations made during previous workshops (W1 and W2) held in India. We noticed that the ideation process sometimes relied too much on existing technologies and did not always address the goal of the activity (i.e., supporting tactile-sensory well-being), a phenomenon also identified in previous research.^46^ Inspired by previous research^47^ that underscores the potential of giving attendees examples to build upon and criticise, we provided attendees with curated, sensory-focused examples as fictive scenarios. This approach kick-started their ideation process and helped them envision new technologies while keeping a focus on sensory well-being. To create these design fiction scenarios, we held a rapid brainstorming session with eight HCI researchers (ages 25 to 40, 3 female, 5 male) from our research group. Engaging in brainstorming sessions with additional researchers helped us break free from our own biases and preconceptions about the future of tactile technologies. The ideation started with presenting key insights from the two workshops conducted in India and workshop 3 in the UK. The researchers then engaged in a rapid brainstorming exercise, generating simple scenarios within 5 min. These scenarios were reviewed and discussed collectively. Finally, we refined and detailed two scenarios that aligned with the workshop findings and created visuals and narratives to depict them, as shown in Figure 3.

***Scenario 1:*** ‘Memory Hourglass’ envisioned a tabletop box that offers a tactile and immersive journey through one’s cherished memories. The hourglass reveals images from the past that users can not only see but also feel, with the sensation of sand slipping through their fingers as the images transition. This idea was inspired by the workshop findings, which highlighted attendees’ joy in cherishing memories and witnessing their families grow. The scenario was designed to be experienced alone or shared with loved ones, reflecting attendees’ appreciation for sharing life’s wisdom.

***Scenario 2:*** ‘Immersive Oasis’ introduced a special pod that transports users to different places through touch and other sensations. For example, it could simulate a calm beach with haptic sensations of wind, the sound of waves, and the scent of the sea. The Immersive Oasis aimed to encourage users to connect with their senses and practice mindfulness meditation in unique, sensory-rich environments. This idea emerged from attendees’ expressed interest in trying new experiences as they aged.

Once these scenarios were presented, the attendees were asked to reflect on these ideas’ potential benefits and drawbacks. They were then prompted with questions such as, ‘Imagine you have a magic wand, and your dream tactile sensation creator comes to life ⋯ What does it do? Where is it? When do you use it? How does it look?’ The attendees envisioned their ideas for about 15 min, then presentations and open discussions. Each attendee shared their envisioned technology, discussing its potential impact on enhancing sensory experiences and emotional well-being.

### Data collection, synthesis and analysis

The workshops were facilitated by a team of three researchers (first three authors) in the UK and a team of two researchers in India (first and fifth authors). The first author moderated all the workshops. Detailed notes were taken individually by each researcher for every group, with additional notes recorded during broader discussions involving all attendees. After each workshop, the researchers convened to discuss and synthesise their individual observations, adding further notes as needed. To ensure accuracy, the notes were cross-checked with visual materials from the workshops, resulting in distinct workshop summaries. All summaries were then compiled, analysed, and discussed iteratively amongst the co-authors.

The analysis initially employed a deductive thematic analysis approach.^48^ The predefined inquiry points from our research guided the high-level coding, which included:

- Attendees’ views on healthy ageing and its challenges.
- Tactile experiences in everyday life.
- Ideas on and expectations from future technologies.

After coding the notes, which was done by Authors 1, 2, and 3, all authors reviewed the codes and themes. We then used an inductive approach to identify any additional patterns or insights by cross-checking with the original workshop notes. Finally, Author 2 summarised the findings, and in a follow-up meeting, we synthesised design implications,^14^ which are presented in the next section.

## Results

Through thematic analysis of the co-production workshops, we compiled our findings from the attendees’ perspectives on healthy ageing, the role of sensory experiences, and the potential for sensory technologies to address these challenges. We also identified cross-cultural differences in perceptions and engagement with technology, which have important implications for designing interventions that are sensitive to diverse cultural contexts.

**Figure 4.**
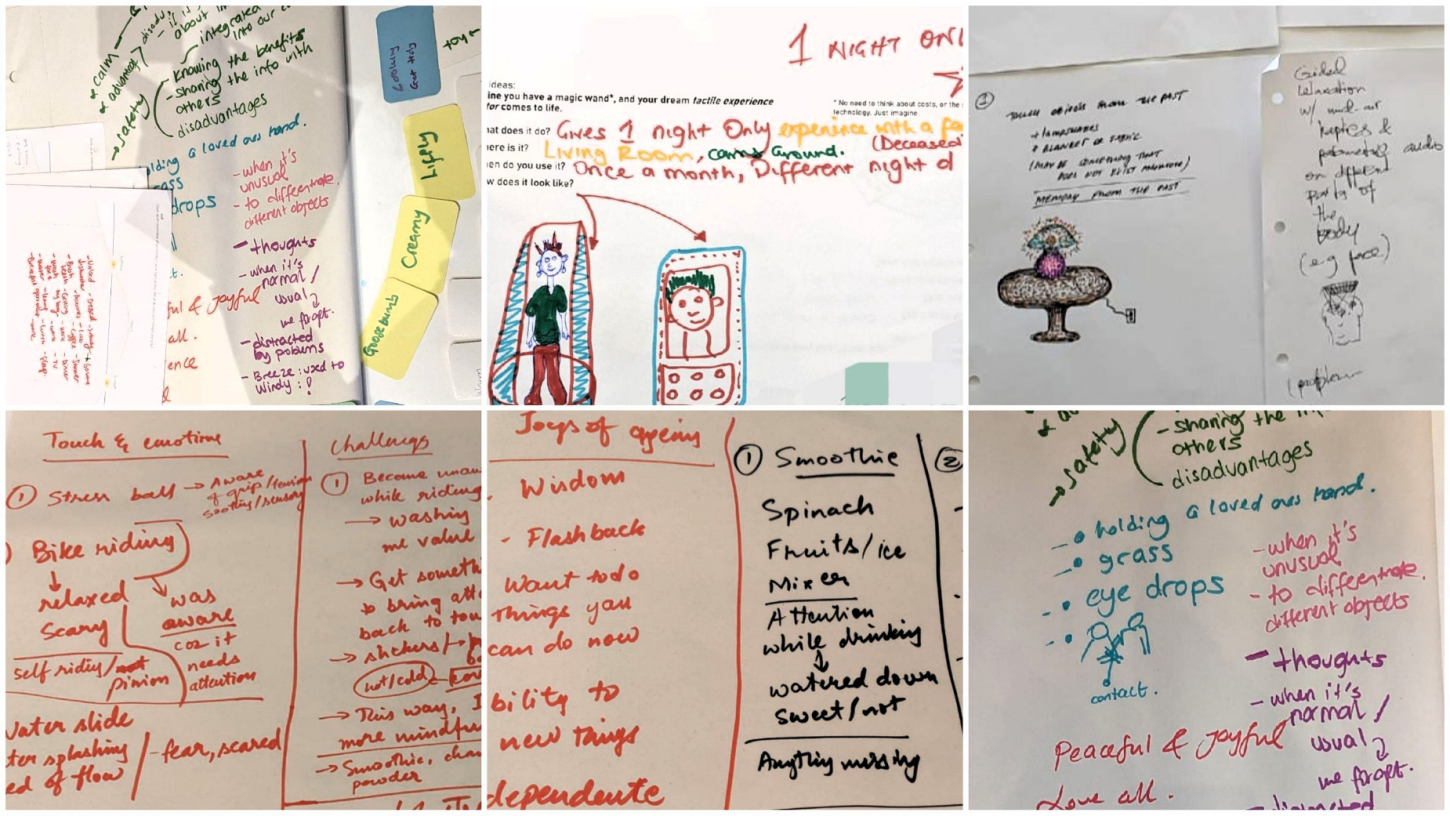
Sample images of notes and ideas on papers by the attendees and the researchers during various phases of the workshops. (Top left) Notes on healthy ageing challenges as described by the attendees, (top middle and right) envisioned ideas sample during W4 by the UK attendees, and (bottom) the notes on everyday sensory experiences and associated emotions.

We began each workshop with a broad discussion on healthy ageing, encouraging attendees to share both the positive aspects and challenges they associate with growing older. These discussions served as a foundation for understanding the needs and priorities of ageing populations and informed the subsequent exploration of sensory experiences and technology design (Figure 4).

### Joys of ageing

Attendees highlighted several positive aspects of ageing, such as increased independence, greater self-confidence, inner tranquillity, and the ability to reflect on a lifetime of experiences. Older attendees especially emphasised the joy of sharing wisdom and guiding others, often drawing satisfaction from watching their families grow. In the workshops held in India, attendees frequently referred to their roles as mentors within their families and communities, as one attendee remarked (translated from a local language to English), “Our life stories and experiences are our biggest assets; we share them to guide our children and grandchildren’. In contrast, UK attendees tended to frame their satisfaction in more individual terms, with a focus on personal fulfilment and pursuing hobbies. A UK attendee noted, ‘Now I finally have time to focus on my passions, without worrying too much about others’. While younger attendees echoed many of these sentiments, they placed less emphasis on family growth and more on personal development and new experiences, particularly in the UK, where attendees expressed a strong desire for personal discovery and independence.

### Challenges to healthy ageing

**Figure 5.**
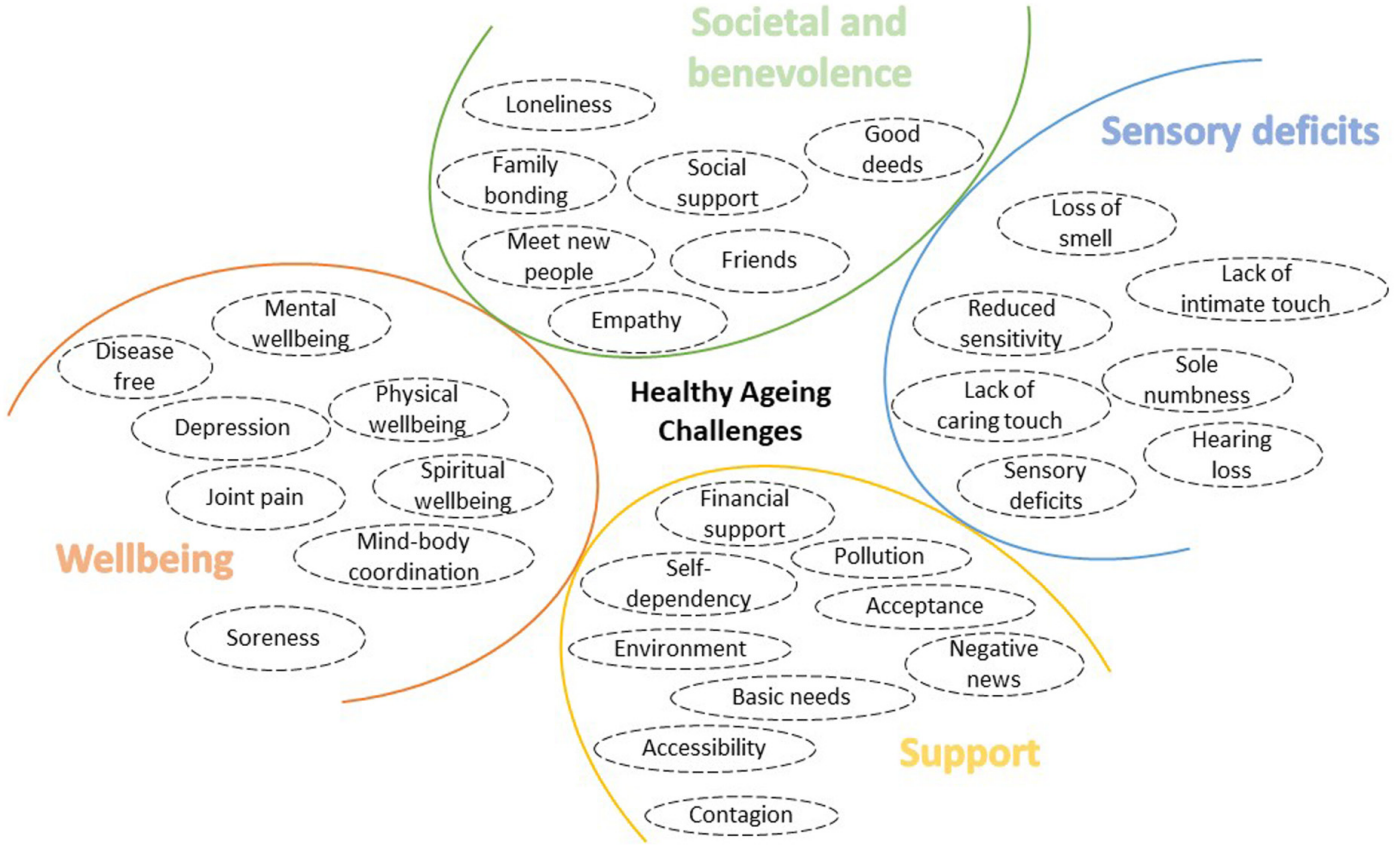
Healthy ageing challenges clustered under four categories: well-being, societal and benevolence, sensory deficits, and support. These challenges served as the foundation for subsequent discussions throughout the remainder of the workshops and were also pivotal in guiding the ideation process for envisioning future sensory interfaces.

Attendees also identified several challenges associated with ageing, which we categorised into four main areas: well-being, societal and benevolence, sensory deficits, and support as illustrated in Figure 5. These challenges underscore the need for tailored interventions that address both the physical and emotional dimensions of ageing.

- **Well-being:** Maintaining physical and mental well-being emerged as a significant challenge, with attendees emphasising the importance of regular exercise, mental engagement, and mindfulness practices to prevent deterioration.
- **Societal and benevolence:** Social isolation and the challenge of maintaining meaningful relationships as one age were common concerns, with attendees noting that decreased social interactions can negatively impact emotional well-being. Despite these challenges, many attendees expressed a strong desire to give back to society through volunteering, mentoring, and civic duties, highlighting the importance of social contribution in later life.
- **Sensory deficits:** Declines in sensory functions, such as tactile sensitivity, hearing, and vision, were frequently mentioned as barriers to maintaining independence and engaging fully with the environment.
- **Support:** Attendees expressed the need for various forms of support to maintain their independence, including physical assistance and accessible living environments.

These challenges set the stage for exploring how sensory experiences in daily life can either alleviate or exacerbate these issues. Understanding the intricate connection between sensory experiences and emotional well-being became the next focal point of our workshops.

### Sensory experiences in everyday activities

**Figure 6.**
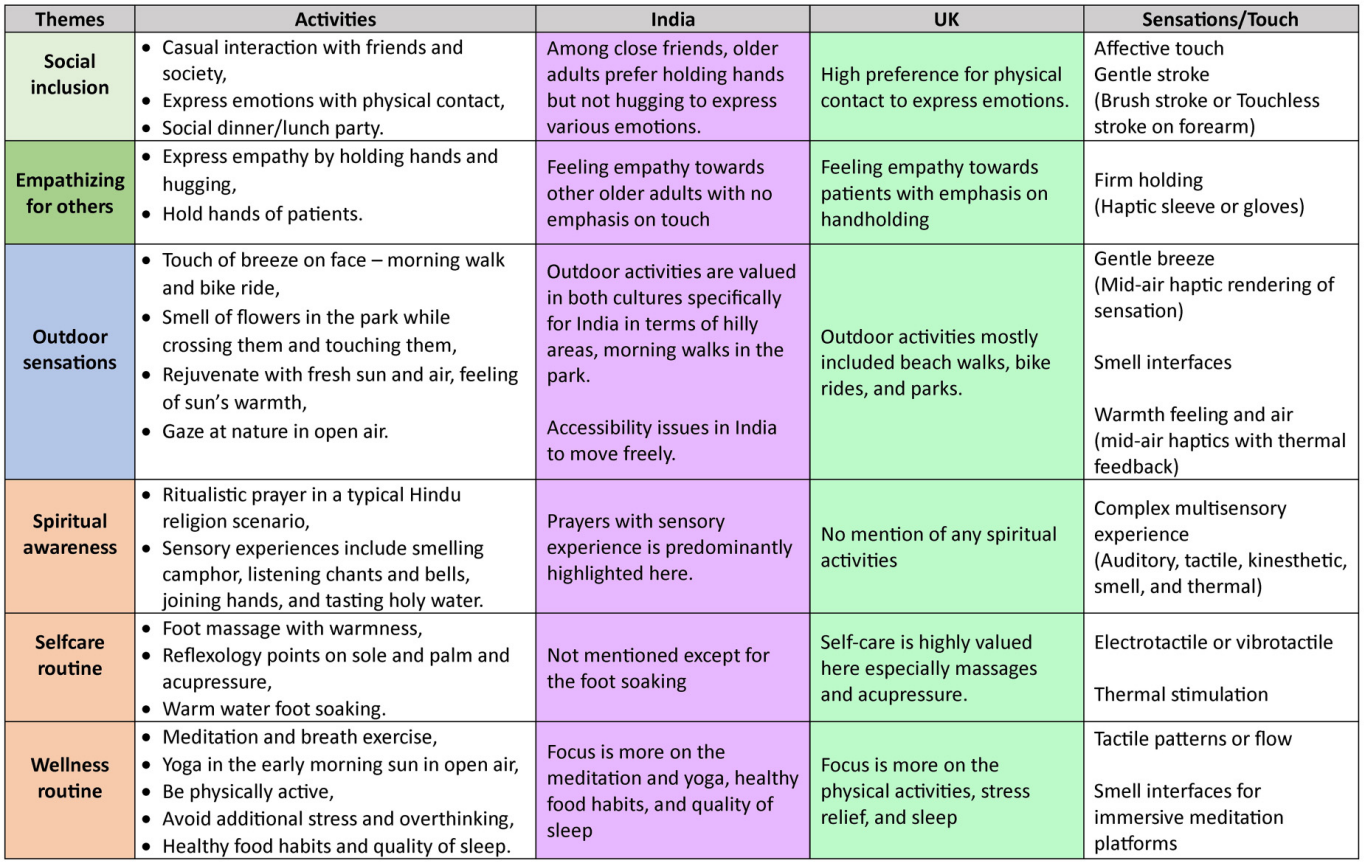
Cross-cultural mapping of everyday sensory activities and associated emotional experiences reported by older adults in India and the UK. Drawing from the clusters given in Figure 5, this table indicates the insights under which the activities reported by the attendees were classified. The first column identifies key thematic clusters from workshop discussions. The second column lists representative activities described by attendees. The third and fourth columns summarises cultural nuances in how these activities are experienced or valued across India and the UK, respectively. The fifth column suggests how current haptic technologies might replicate or support these sensory experiences. This mapping provides a foundation for culturally responsive design of tactile technologies for healthy ageing.

Attendees reflected on the sensory dimensions of their everyday activities, revealing how deeply intertwined sensory experiences are with emotional well-being. Six key themes emerged from these discussions: social inclusion, empathising with others, outdoor sensations, spiritual awareness, self-care routines, and wellness routines. Figure 6 summarises these themes, showing the cultural nuances and how current haptic technologies might replicate these sensory experiences.

- **Social inclusion:** Social interactions were closely linked to sensory experiences, with attendees in India often associating them with extended family gatherings, where touch played a significant role, such as the feeling of a loved one’s hand or the warmth of a hug. These physical sensations were integral to expressing emotions and fostering connection. In contrast, UK attendees highlighted smaller social clubs and gatherings with friends, where sensory interactions such as the feel of a handshake or a pat on the back were common ways to show camaraderie and emotional support. These tactile experiences of touch and proximity helped reinforce social bonds and provided comfort, emphasising the sensory dimension of social inclusion across both cultures.
- **Empathising with others:** Sensory experiences were also tied to empathy, particularly through touch, as attendees described emotionally charged moments such as holding a loved one’s hand or offering comfort to someone in need.
- **Outdoor sensations:** Outdoor activities were rich in sensory experiences, with attendees enjoying the feel of wind, sun, and natural elements. Indian attendees often connected these sensations to spiritual practices, while UK attendees emphasised recreational activities such as gardening and beach walks.
- **Spiritual awareness:** Spiritual practices were a significant source of sensory experiences, especially among Indian attendees who described rituals involving touch, smell, and sound. UK attendees who mentioned spirituality often related it to quiet reflection or meditation.
- **Self-care routines:** Sensory-rich self-care activities such as massages, bathing, and personal grooming were highlighted by attendees in both countries, though framed in different cultural contexts. Indian attendees linked self-care to holistic well-being, while UK attendees emphasised personal relaxation.
- **Wellness routines:** Daily wellness practices, including stretching, meditation, and dietary rituals, were sensory in nature and contributed to attendees’ overall well-being. Indian attendees connected these routines to traditional practices such as yoga, while UK attendees focused on physical activities such as walking.

Figure 6 provides a detailed look at these themes, showing how they differ between the two countries and how they could be supported by current haptic technologies.

### Challenges in tactile sensation awareness

Despite the richness of these sensory experiences, attendees identified several challenges in maintaining tactile awareness. Habituation to familiar experiences, stress-induced insensitivity, and environmental distractions were common obstacles. These challenges were consistent across age groups and geographical locations, underscoring the need for interventions that can help individuals remain mindful of their sensory experiences, even in the face of stress or routine.

### Cross-cultural insights

Cross-cultural differences also emerged in how attendees approached mindfulness, ageing, and technology. UK attendees often emphasised the cognitive benefits of mindfulness, while Indian attendees aligned mindfulness with spiritual experiences. These differences reflect broader cultural attitudes towards ageing and well-being, which must be considered when designing technologies for diverse populations.

- UK attendees emphasised independence, self-discovery, and personal fulfilment, while Indian attendees highlighted the importance of familial bonds and collective well-being.
- Emotion-tactile connections were universal across both cultures, though UK attendees focused more on modern life experiences, whereas Indian attendees drew on traditional practices and rituals.
- Reactions to mid-air haptic technology also differed, with UK attendees being more open to its integration, while Indian attendees expressed caution, reflecting cultural differences in attitudes towards new technologies.

### Attendees’ ideas on future technologies for tactile and sensory well-being

**Figure 7.**
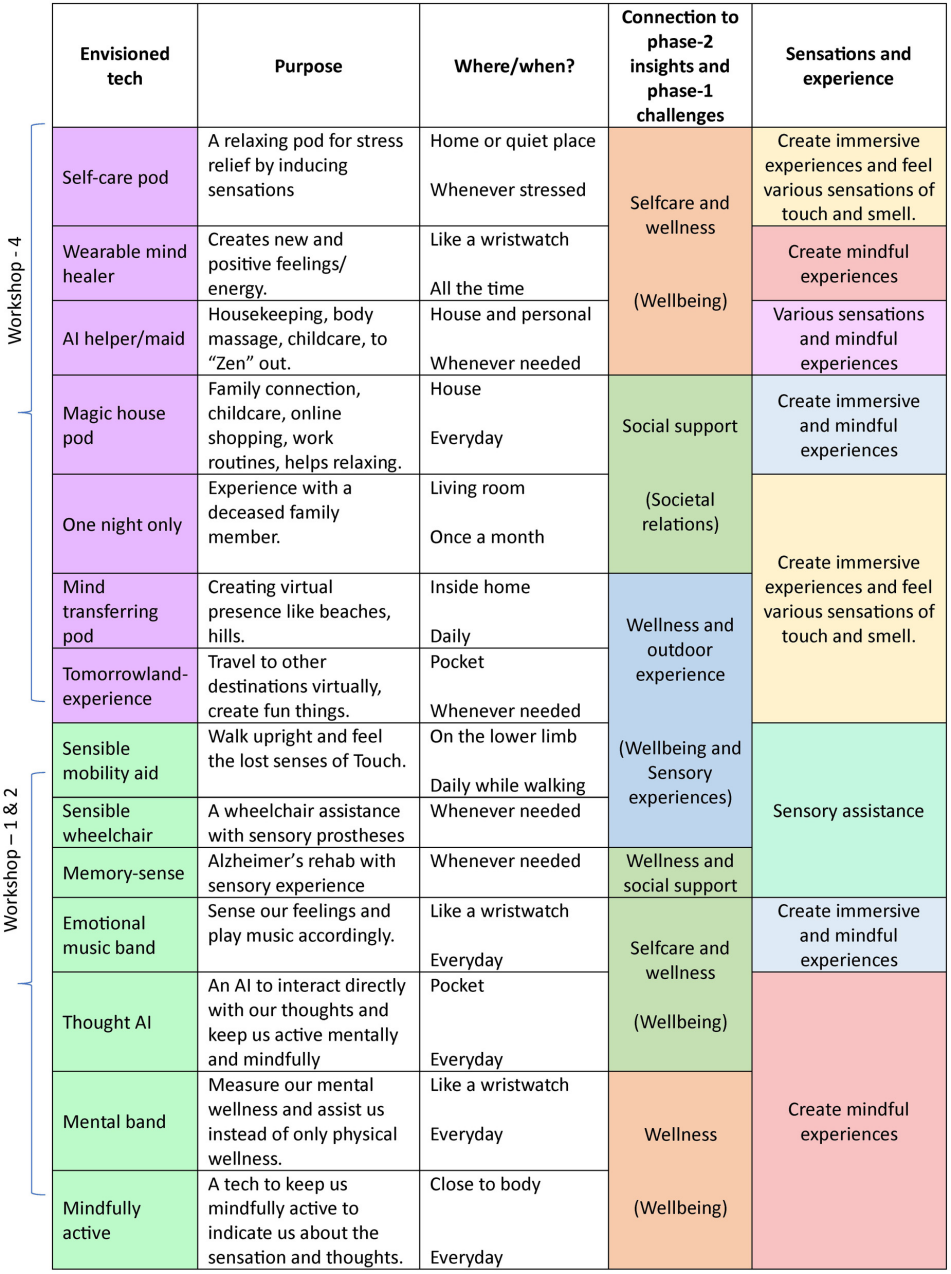
Envisioned technologies created by attendees during the workshops, categorised based on the purpose they serve and the associated sensory experiences. These ideas reflect a wide range of aspirations, from self-care and emotional well-being to immersive and mindful sensory interactions. The figure shows the clustering of ideas, derived from phase-2 discussions, and highlights the cross-cultural nuances between the UK and India. Note that W3 (UK Part 1) did not involve the envisioning phase, as it focused primarily on exploring sensory experiences and emotional connections, reserving the envisioning for W4 (UK Part 2) to allow more in-depth reflection and ideation.

During the final phase of the workshops, attendees were invited to envision future technologies that could address the challenges of healthy ageing and enhance sensory experiences. Drawing on their discussions of sensory decline, mindfulness, and emotional well-being, attendees generated a diverse range of ideas that were both practical and imaginative. These ideas are visualised in Figure 7, where attendees’ contributions are categorised according to their purpose and the sensory experiences they aim to support.

Additionally, there was strong interest in **assistive wearables** that would promote sensory awareness and emotional regulation. Attendees proposed wearable devices, such as wristbands, that provide calming tactile feedback during stressful moments. These devices could also monitor physiological signals such as heart rate, responding with soothing vibrations or pulses to enhance relaxation.

In the workshops held in the UK, attendees placed particular emphasis on **memory enhancement tools**, technologies designed to evoke cherished memories through tactile and sensory feedback. One notable idea was a device that could recreate the feeling of holding a loved one’s hand, using haptic sensations to trigger emotional and sensory memories from the past.

Building on discussions around mindfulness, attendees also proposed **mindfulness and meditation aids**. These tools would incorporate subtle tactile feedback to help individuals maintain focus during meditation, such as gentle haptic prompts to guide breathing or body awareness.

Finally, several ideas centred on **empathy and connection technologies** that could enhance emotional bonds across distances. Attendees envisioned devices that would allow users to share tactile sensations with loved ones in real-time, fostering a sense of emotional closeness even when physically apart.

These envisioned technologies, mapped in Figure 7, not only address the functional challenges of sensory decline but also emphasise the emotional and relational aspects of sensory experiences in ageing populations. The ideas reflect a desire for innovations that enhance sensory immersion, well-being, and social connectedness.

These ideas showcase the attendees’ desire to harness technology not only to address the physical challenges of ageing but also to enrich their emotional and sensory lives. The envisioned technologies emphasise the potential for innovation to create meaningful and supportive experiences that can enhance well-being, strengthen social bonds, and promote mindfulness in older adults. These insights will inform future design efforts, ensuring that technological solutions are grounded in the real needs and aspirations of ageing populations.

The workshops also provided insights into attendees’ experiences with mindfulness meditation and their interactions with mid-air haptics. These insights further inform the design of future technologies that support sensory well-being. While few attendees had prior experience with mindfulness meditation, those who did shared mixed responses. Initial difficulties with environmental factors, such as lighting and seating, gradually gave way to a deeper engagement with the practice. Attendees collectively defined mindfulness as a state of present-centeredness, free from distractions, characterised by heightened awareness of surroundings, thoughts, and emotions.

### Engagement with mid-air haptic technology

Attendees interacted with mid-air haptic technology during the workshops, and age-based differences in responses emerged. Younger attendees were more open to the technology, expressing fewer safety concerns. In contrast, older attendees were more sceptical, emphasising the need for clear communication about the benefits and safety of new technologies. While digital literacy^49^ may have played a role in these differences, we did not collect data on this, limiting our ability to assess its influence. Future research should consider digital literacy alongside age when introducing emerging technologies. This highlights the importance of considering digital literacy in future research and the need for context-sensitive communication when introducing emerging technologies to older adults.

In sum, the workshop findings reveal diverse perspectives on healthy ageing, the centrality of sensory experiences to well-being, and the potential for mindfulness and technology to enhance the lives of older adults. These insights underscore the importance of culturally sensitive, context-aware approaches to designing future technologies for healthy ageing.

## Discussion

The insights from our co-production workshops in India and the UK provide valuable, qualitative themes that inform our understanding of sensory experiences, ageing, and the potential role of technology in enhancing the well-being of older adults. These findings build upon existing literature in HCI, ageing, and sensory engagement, while offering a nuanced perspective on how technology can be designed in an age-specific and culturally relevant way. Notably, the workshops revealed cross-cultural differences in the sensory experiences and needs of older adults, underscoring the importance of contextually sensitive designs. Here we first discuss and reflect upon these findings and insights, highlighting the cross-cultural nuances between the two geographic locations. We then outline four key design implications^14^ to inform HCI research and practice pertaining to digital health. These implications underscore the need for digital health interventions that leverage mindfulness and sensory technologies to address age-related sensory decline. Such tools could be deployed in remote monitoring systems or therapeutic platforms to enhance emotional and cognitive health.

### Cross-cultural distinctions

**Figure 8.**
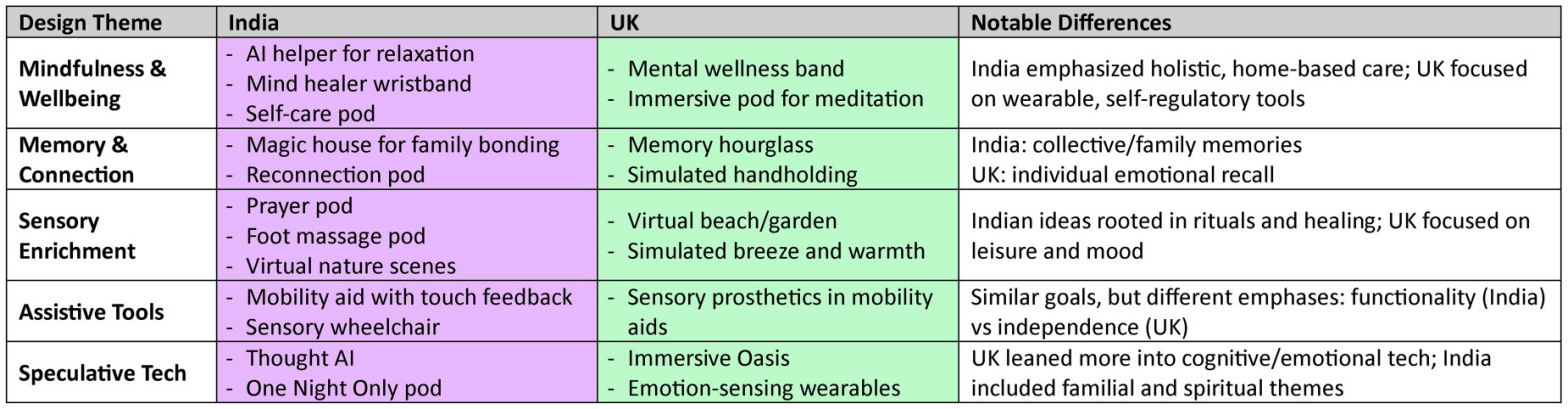
Summary of design ideas across India and the UK, grouped by design themes. The table highlights commonalities and observed differences in how older adults from each cultural context envisioned future technologies to support sensory well-being, mindfulness, memory, and assistive needs. It also includes interpretations that reflect the influence of cultural values and practices on attendees’ preferences and expectations from tactile and digital health technologies.

Our co-production workshops revealed meaningful cultural distinctions in how older adults in India and the UK experience touch, mindfulness, and ageing. Indian attendees emphasised collective well-being, mentorship to younger generations, and spiritually grounded sensory rituals. In contrast, UK attendees highlighted personal autonomy, emotional self-regulation, and individualised self-care routines (Figure 8). These patterns align with broader cultural tendencies described in frameworks such as Hofstede’s cultural dimensions theory,^32^ which identifies key dimensions where cultures may differ, including individualism versus collectivism and hierarchical versus egalitarian structures. While Hofstede’s model provides a useful lens for interpreting these findings, we acknowledge that it is not the only framework for understanding cultural influences.^33^ Our choice to apply this approach was guided by its widespread use in cross-cultural HCI research and its relevance in identifying patterns that emerged from our data. These cultural orientations help explain how older adults in each context prioritise family connections or personal independence in shaping their sensory and emotional well-being.

Similarly, perceptions of emerging technologies such as mid-air haptics reflected differing cultural attitudes towards novelty and risk. UK attendees were more exploratory and open in their engagement with speculative design scenarios, reflecting a cultural orientation that embraces innovation and long-term self-improvement. Indian attendees, while interested, approached these technologies with more caution and emphasis on safety, which aligns with cultural tendencies that value tradition and lower tolerance for ambiguity. Although we note that digital literacy may also play a role in these differences, it was not directly measured in this work. These cultural differences in the perception of mid-air haptics may also be influenced by the diverse socio-economic and educational backgrounds of attendees, as detailed in the ‘Workshops context and background’ section. Applying Hofstede’s framework provides a valuable lens for understanding how cultural values influence both ageing experiences and expectations from technology, reinforcing the need for culturally responsive design in digital health.

### Integrating mindfulness into sensory technology design

The first design implication and one of the central findings from the workshops is the potential for integrating mindfulness practices into sensory technology design. While existing research in HCI has explored sensory substitution,^28,29^ the emphasis has often been on compensating for sensory loss. Our workshops suggest that a more holistic approach, where mindfulness is embedded into haptic interactions, can not only address sensory decline but also actively enhance emotional and physical well-being. This aligns with recent studies that have highlighted how mindfulness meditation can heighten sensory perception and improve emotional states.^22,23^ Virtual reality-based mindfulness interventions have demonstrated initial psychological and physiological health benefits, including reduced stress and improved emotional regulation, but require more rigorous and long-term studies to validate these effects.^50^ Adapting mindfulness-based programs^51^ to specific populations and contexts, as outlined by frameworks for targeted modifications, ensures that digital mindfulness interventions are not only effective but also culturally and contextually relevant for diverse user groups (e.g. older adults).

### Fostering social affective interactions

The second design implication centres on fostering social affective interactions, particularly to combat loneliness and strengthen social bonds among older adults. Attendees in both India and the UK highlighted the importance of touch in maintaining emotional connections, whether through holding hands with a loved one or participating in family gatherings. While HCI has long recognised the importance of social interaction in the design of assistive technologies,^30^ our findings suggest that tactile, social interactions – such as recreating the comforting sensation of a hug or handshake – can play a crucial role in enhancing emotional well-being.

This implication is particularly relevant in culturally diverse contexts. In India, social interactions were often tied to family and spiritual practices, whereas in the UK, attendees emphasised personal well-being and individual social networks. Technologies designed to foster these interactions should be adaptable to different cultural contexts, supporting both individual and collective forms of well-being. This echoes prior research that emphasises the role of culture in shaping how older adults interact with technology.^31^

### Designing for sensory immersion indoors

The third design implication and a key finding from our workshops is the potential for sensory technologies that bring outdoor sensations indoors, particularly for older adults who may face mobility limitations or other barriers to accessing natural environments. Attendees in both countries expressed a desire for technologies that could simulate the sensory richness of outdoor experiences, such as the warmth of the sun or the feel of a gentle breeze. This finding is consistent with previous research that highlights the importance of nature-based sensory experiences for emotional and physical well-being in older adults.^19^

While the general desire for sensory immersion was consistent across cultures, the specific applications differed. UK attendees focused on leisure and recreational activities, such as simulated walks on the beach or time spent in gardens, whereas Indian attendees often associated outdoor sensations with spiritual practices, such as morning prayers conducted in natural settings. Similar to the previous implication, this cultural nuance suggests that sensory technologies should be designed with flexibility in mind, allowing users to customise the experiences according to their personal and cultural preferences.

### Addressing sensory decline through adaptive, customisable technologies

Finally, as the fourth design implication, our workshops highlighted the need for adaptive and customisable technologies that can address sensory decline while remaining relevant to users’ evolving needs. As tactile sensitivity, hearing, and vision diminish with age,^10,27^ older adults require technologies that can adjust to these changes. Our attendees expressed a desire for technologies that not only compensate for sensory deficits but also provide enriching, meaningful experiences that enhance their quality of life.

For instance, customisable haptic feedback could allow users to adjust the intensity or type of sensory stimulation they receive, ensuring that the technology remains effective as their sensory abilities change over time. This approach builds on existing HCI research on sensory substitution and assistive technologies,^28^ but it emphasises the importance of ongoing engagement with the technology, rather than a one-time solution. Such flexibility ensures that these technologies continue to support well-being in the long term. For example, scent-delivery devices combined with digital healthcare technologies have shown promise in olfactory training for Parkinson’s disease patients, enabling potential improvements in scent function and earlier diagnosis of neurodegenerative conditions through user-centred HCI designs.^52^

These four design implications underscore the importance of culturally sensitive design in HCI. While much has been done to explore the role of culture in technology adoption,^31,40^ our findings suggest that further work is needed, particularly in the context of sensory experiences and mindfulness. Technologies for older adults should not only address sensory decline but also resonate with the cultural values and practices of the users. For example, while UK attendees valued personal independence and self-care, Indian attendees placed a stronger emphasis on family connections and spiritual practices. These insights highlight the need for technologies that can cater to both individual and collective forms of well-being, depending on the cultural context. Furthermore, tactile and sensory technologies hold significant therapeutic potential for age-related conditions such as dementia and depression by enhancing cognitive engagement, reducing agitation, and fostering emotional connection through innovative tools such as haptic feedback and multisensory environments. Furthermore, integrating these technologies into remote care systems and digital therapeutics can enable real-time monitoring of sensory health, support emotional well-being, and complement behavioural health interventions to improve the quality of life in older adults.

### Limitations and future directions

While our workshops provide valuable insights into the relationship between sensory experiences, emotional well-being, and ageing, it is not without limitations. First, the scope of this research was limited to workshops conducted in two countries, India and the UK. Although these locations provided contrasting cultural perspectives, the findings cannot be generalised to other regions. The cross-cultural nuances in ageing, mindfulness, and sensory experiences may vary significantly in other parts of the world.

Future research will aim to address this limitation by extending our work to other cultural contexts. Given that the authors come from diverse backgrounds, including China, Turkey, and Italy, we plan to leverage these connections to explore the role of touch and sensory experiences in ageing across different societies. Notably, Italy has one of the highest elderly populations in the world, second only to Japan. This makes it an ideal context for further exploration of ageing and sensory engagement, allowing us to develop a more comprehensive understanding of how different cultures approach sensory experiences and mindful technologies for older adults. Future work should incorporate principles of responsible innovation to ensure that emerging mid-air haptic systems align with societal values and address potential ethical challenges. Recent work has highlighted the importance of integrating anticipation, inclusivity, and reflexivity to guide the design of future touchless technologies, particularly in sensitive social scenarios.^53^

Additionally, our work focused primarily on the ideation phase, where attendees contributed their ideas for future technologies. In future work, we will move beyond ideation to prototype and evaluate these technologies in real-world settings. By testing the proposed mindful touch technologies with older adults in everyday environments, we can assess their impact on well-being and refine the designs based on user feedback. Quantitative assessments such as vibrotactile perception threshold measurements^54^ could be used to evaluate sensory function and detect early signs of sensory decline in ageing populations, as demonstrated in previous work on diabetic peripheral neuropathy.^55^ In parallel, physiological indicators such as breath rate variability can be employed to study the short-term effects of these technologies on emotional regulation and mindfulness performance during interaction.^56,57^ Such multimodal approaches will support the development of inclusive digital health solutions that are grounded in both sensory feedback and emotional well-being, as emphasised in recent work on emerging technologies and their societal relevance.^58^

Finally, our workshops primarily explored tactile sensory experiences. Future research could expand to include other sensory modalities, such as auditory, visual, and olfactory experiences, to provide a more holistic understanding of how multiple senses interact to shape emotional well-being in ageing populations.

## Conclusion

In this paper, we explored the intersection of sensory engagement, mindfulness, and digital technology to address the challenges of healthy ageing. Through co-production workshops in India and the UK, we identified key themes around sensory decline, emotional well-being, and the potential of mindful technologies to enhance the lives of older adults. We presented four key design implications highlighting the importance of integrating mindfulness into haptic technologies, fostering social affective interactions, and enabling sensory immersion for ageing populations. Furthermore, our work underscores the need for culturally sensitive and customisable solutions that cater to diverse experiences of ageing. By leveraging these insights, future research and HCI practices can develop technologies that not only compensate for sensory loss but also enrich emotional and tactile experiences, promoting holistic well-being for older adults. These contributions set the stage for the design of inclusive, meaningful innovations in the field of ageing and sensory-powered digital health technology.
